# Toxicity and efficacy of Gamma Knife radiosurgery for brain metastases in melanoma patients treated with immunotherapy or targeted therapy—A retrospective cohort study

**DOI:** 10.1002/cam4.3021

**Published:** 2020-04-06

**Authors:** Brigitte Gatterbauer, Dorian Hirschmann, Nadine Eberherr, Helena Untersteiner, Anna Cho, Abdallah Shaltout, Philipp Göbl, Fabian Fitschek, Christian Dorfer, Stefan Wolfsberger, Gregor Kasprian, Christoph Höller, Josa M. Frischer

**Affiliations:** ^1^ Department of Neurosurgery Medical University Vienna Vienna Austria; ^2^ Department of Biomedical Imaging and Image‐guided Therapy Medical University Vienna Vienna Austria; ^3^ Department of Dermatology Medical University Vienna Vienna Austria

**Keywords:** brain metastases, gamma knife radiosurgery, immunotherapy, melanoma, targeted therapy

## Abstract

**Background:**

Few safety data of concurrent stereotactic radiosurgery and targeted therapy (TT) or immunotherapy (IT) are available. The aim of the study was to evaluate the outcome of melanoma patients with brain metastases (MBM) after Gamma Knife Radiosurgery (GKRS) in relation to IT/TT.

**Methods:**

We evaluated 182 MBM patients, who were treated with GKRS in the modern radiosurgical and oncological era.

**Results:**

The median time between the initial melanoma diagnosis and occurrence of MBM was 2.4 years. The median overall survival time was 5.4 years after melanoma diagnosis. The estimated median survival after the initial diagnosis of MBM was 1.0 year (95% CI = 0.7‐1.2 years). Patients treated with anti‐PD‐1 or a combination of anti‐CTLA‐4/PD‐1 showed a significantly longer survival after first GKRS compared to all other forms of treatment. In addition, patients treated with anti‐PD‐1, anti‐CTLA‐4, or a combination of anti‐CTLA‐4/PD‐1 showed a significantly longer time to new MBM after GKRS1 compared to patients treated with other forms and combinations of the oncological therapy. The occurrence of hemorrhage or radiation reaction/necrosis after GKRS did not show any statistically significant differences in relation to IT/TT.

**Conclusion:**

In MBM patients, complications after GKRS are not significantly increased if IT/TT treatment is performed at the time of or after radiosurgery. Further, a clear benefit in distant control and survival is seen in MBM patients treated with GKRS and checkpoint inhibitors. Thus, concomitant treatment of MBM with GKRS and IT/TT seems to be a safe and powerful treatment option although further prospective studies should be conducted.

## Graphical abstract

In melanoma brain metastases patients, complications after GKRS are not significantly increased if IT/TT treatment is performed at the time of or after radiosurgery, and a clear benefit in distant control and survival is seen. Thus, concomitant treatment of MBM with GKRS and IT/TT seems to be a safe and powerful treatment option.

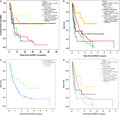

## 1 BACKGROUND

Over the last decades, the incidence of melanoma has been rising, representing the third most common cause of brain metastases.^1^, ^2^ Survival in advanced disease stages has improved significantly over the last 10 years, following the approval of immunotherapy with check‐point inhibitors and targeted therapy with BRAF & MEK inhibitors.^3^ Patients with asymptomatic brain metastases seem to benefit from these novel therapeutic standards, especially from the combined immune‐checkpoint inhibition with Ipilimumab and Nivolumab.^4^, ^5^, ^6^ Local treatment options for melanoma patients with brain metastases (MBM) are surgery, stereotactic radiosurgery, fractionated radiotherapy, and whole brain irradiation (WBRT). Since MBM have shown to be rather resistant against the radiotherapy and cytotoxic chemotherapy, the treatment of especially symptomatic MBM remains a challenge.^5^, ^7^, ^8^

Gamma Knife radiosurgery (GKRS) allows the delivery of a high dose to the lesion with a rapid radiation fall off to the surrounding normal brain parenchyma, resulting in high tumor control rates.^9^ In addition, the risk of neurocognitive side effects is significantly reduced compared to WBRT.^10^ Historically, radiosurgery was only recommended for patients in a good performance status with few lesions. Nowadays, patients with 10 metastases and more are regularly treated by GKRS.^11^ In recent years, development and utilization of new oncological therapies increased the overall survival of melanoma patients but has further led to discussions about the safety of the concurrent stereotactic radiotherapy (SRT) and TT or immunotherapy. So far, available data are scarce. In this retrospective cohort study we assessed the radiological and clinical outcome data of patients with MBM, who were treated in the modern radiosurgical and oncological era.

## 2 METHODS

### 2.1 Patient sample and data evaluation

Since 1992, patients with brain metastases have been treated with GKRS at our department. For the evaluation of the modern radiosurgical treatment in relation to the modern oncological therapy, a retrospective analysis of 182 MBM patients treated between 2012‐2018 was performed. Clinical data from our EDP system were used. Treatment with IT or TT was evaluated separately for two different time periods: at the time of radiosurgical treatment (±30 days) and after radiosurgical treatment (>30 days). Patients were further grouped into those who never received IT/TT at or after GKRS1 and those who did receive IT/TT at or after GKRS1. Since we evaluated concomitant IT or TT and GKRS we did not account for the oncological treatments longer than 30 days prior to GKRS1. Additional treatments, such as corticosteroids, were evaluated as well. Furthermore, patients were assessed using the GPA (Graded Prognostic Assessment; general and specific for primary tumors), RPA (Recursive partitioning analysis), and SIR (Score Index for Radiosurgery).^12^, ^13^, ^14^ To minimize the selection bias, all patients were included in the primary analysis. The study was approved by our institutional review board. Since analysis of data were done retrospectively, patient consent was not obtained.

### 2.2 Radiosurgical technique

Patients were treated with a Leksell Gamma Knife Perfexion^®^ (Elekta AB, Stockholm, Sweden). Gamma Plan (Elektra AB) was used as the planning software. MRI planning sequences were performed on a 1.5‐T magnet (Philips Ingenia; Philips Healthcare) using an eight channel head coil. Axial and coronal gadolinium contrast‐enhanced T1‐weighted MRI sequences and multiplanar T2‐weighted sequences were used in treatment planning. The target was defined as a contrast‐enhanced tumor mass on T1 sequences and as a hypointense tumor mass on T2 sequences. The whole tumor mass was covered without an additional margin. All metastases visualized on planning MRI were treated with GKRS. When new MBM were detected during follow‐up MRI, another GKRS was the therapy of choice in most cases as long as the patient´s clinical condition allowed it. Thus, a total of 868 MBM were treated in 288 radiosurgical procedures. A median number of 2 MBM were irradiated per GKRS treatment but varied with a wide range of 1‐16 MBM. The majority (109/182, 60%) of patients underwent one Gamma Knife treatment.

A third of patients (54/182, 30%) received a second GKRS while the remaining patients (19/182, 10%) received up to six radiosurgical treatments. Multiple GKRS were mainly performed due to newly diagnosed MBM. In 12% of patients (21/182, 12%) larger MBM in eloquent localizations were treated by two‐fraction dose‐staged GKRS as described before.^15^ Treatment planning mainly occurred on the 50% isodose line (40%‐90%), with a median prescription dose of 20 Gy (6‐20Gy), and a median central dose of 33 Gy (12‐50Gy). The median treatment volume was 0.7 ccm, with a wide range of 0.1‐19.4 ccm.

### 2.3 Follow‐up and outcome evaluation

**Figure 1:**
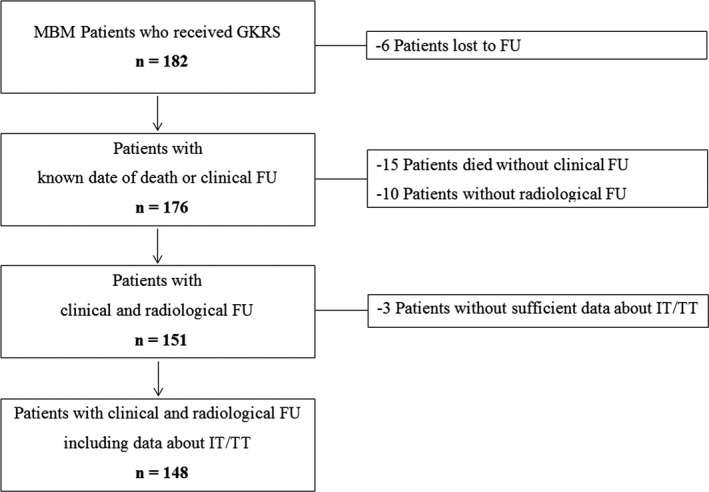
Flow chart depicting study inclusion algorithm. About 182 melanoma patients with brain metastases were treated with GKRS between June 2012 and December 2018 at our institution. After GKRS, patients were routinely followed in a 3‐month interval with brain MRI and a clinical assessment according to our standard procedure. However, as often seen in clinical routine, not every patient adhered to this schedule. Patients who were lost to follow‐up were included in the study but excluded from the complication analyses. In addition, we performed a death register comparison using data provided by Statistik Austria^©^. At the time of study conclusion, the majority of patients (121/182, 67%) had already succumbed to their disease. Thus, only six patients (6/182, 3%) were truly lost to follow‐up. Twenty‐five patients (25/182, 14%) died prior to or without a radiological or clinical follow‐up. Consequently, they had to be excluded from our outcome analyses regarding complications. Thus, we are able to provide survival analyses on 176 MBM patients and complication analyses on 151 patients. Survival analysis was also performed in reference to IT/TT. The majority of our patients (148/176, 84%) received immunotherapy or targeted therapy (IT/TT) at the time of their first radiosurgical treatment or after GKRS1. Twenty patients (20/176, 11%) had never received any IT/TT at or after GKRS1. Eight patients (8/176, 5%) did not receive any IT/TT at GKRS1, but since no clinical follow‐up was available, we had to exclude them from the outcome analysis in relation to IT/TT

After GKRS, patients were routinely followed in a 3‐month interval with brain MRI and a clinical assessment according to our standard procedure. However, as often seen in clinical routine, not every patient adhered to this schedule. Patients who were lost to follow‐up were included in the study but excluded from the complication analyses (Figure 1). Complications after radiosurgery were systematically evaluated on follow‐up MRIs. Based on standard MRI sequences (FLAIR/T2‐weighted, T1w pre‐ and post‐gadolinium based contrast agent, and DWI sequences) and according to the RANO criteria, progression was defined as at least 20% increase in the longest diameter.^16^ Radiation reaction was defined as progressive surrounding edema, while radiation necrosis, in general, was defined as a progressive ring‐enhancing lesion with surrounding edema.^17^ Intralesional hemorrhage was either identified as novel intralesional increase of CT density, compatible with hemorrhage or progressive or novel additional (T2‐weighted or T2*‐weighted hypointense or T1 hyperintense) signal alteration.

### 2.4 Statistical analysis

Due to the uneven distribution of data, nonparametric tests were used. Descriptive analyses included median values and ranges. Statistical calculations included the chi‐square test; the Kaplan‐Meier method and actuarial life‐table analysis were used to estimate the progression as well as the survival rates. Differences between groups were assessed by the Logrank or Breslow test as appropriate. Survival in relation to the prognostic scores was evaluated by pairwise Wilcoxon test. *P*‐values < .05 were considered to be statistically significant. IBM SPSS Statistics for Windows (Version 25.0 Armonk, NY: IBM Corp.) was used for all statistical calculations.

## 3 RESULTS

### 3.1 Patient presentation and overall survival

At the time of MBM diagnosis, the vast majority of patients (170/182, 93%) had already been diagnosed with extracranial metastases (Table 1). Thus, the majority of patients (160/182, 88%) were rated as RPA class II; six patients (6/182, 3%) were classified as RPA class I, and 16 patients (16/182, 9%) were classified as RPA class III. The median time between the initial melanoma diagnosis and occurrence of MBM was 2.4 years (0.0‐23.0 years). Patients with occult melanoma (0.5 years, 0.0‐5.8 years) presented with MBM significantly earlier compared to cutaneous (2.7 years, 0.0‐21.7 years; *P *= .001) or uveal melanoma patients (20.6 years, 4.1‐23.0 years; *P *= .007). The median overall survival time after diagnosis of the primary tumor among all patients was 5.5 years (95% confidence interval [CI] = 4.5‐6.5 years). According to the Logrank comparison of estimated survival, there was a significant difference in overall survival among melanoma subtypes (*P *= .012): Uveal melanoma patients presented with the longest survival followed by cutaneous melanoma patients.

**Table 1:** Sample characterization

	Time of first GKRS—total sample (n = 182)
Age	64
In years, median (range)	(25‐89)
Female: male ratio	71:111
KPS	90
In %, median (range)	(40‐100)
ECM Status at time of BM diagnosis	
ECM present	170 (93%)
No ECM	12 (7%)
Melanoma subtype	
Cutaneous melanoma	154 (84%)
Uveal melanoma	3 (2%)
Occult melanoma	22 (12%)
Mucosal melanoma	3 (2%)
BRAF status	
Mutated	103 (56%)
Wild type	63 (35%)
Not known	16 (9%)
CNS treatment before GKRS1	
None	149 (82%)
WBRT or fRT	9 (5%)
BM resection without RT	18 (10%)
BM resection with WBRT and/or fRT	6 (3%)
Localization of MBM at initial diagnosis	
Multiple	100 (55%)
Frontal	24 (13%)
Parietal	13 (7%)
Temporal	7 (4%)
Occipital	13 (7%)
Central	11 (6%)
Basal ganglia/ brainstem/other	5 (3%)
Cerebellar	9 (5%)
Predicted survival after prognostic scores *in months, median (range)*	
GPA general	3.8 (2.6‐11.0)
GPA specific	8.8 (3.0‐13.2)
RPA	4.5 (2.3‐7.7)
SIR	6.0 (2.1‐8.8)

Note

The table depicts patient sample characterization at the time of first GKRS for the total sample. Prior CNS treatment was mainly performed for distant brain metastases (BMs). We evaluated the Graded Prognostic Assessment (GPA general and specific), recursive partitioning analysis (RPA), and the Score Index for Radiosurgery (SIR) for each patient.

Abbreviations: ECM, extracranial metastases; fRT, fractionated radiotherapy; GKRS, Gamma Knife radiosurgery; KPS, Karnofsky Performance Status Scale; WBRT, whole brain radiation therapy.

### 3.2 Local tumor control and radiological follow‐up after radiosurgery in relation to immunotherapy and targeted therapy

The median follow‐up after GKRS1 was 0.6 years (range: 0.02‐6.6 years) with a total amount of 210.2 follow‐up years. At the time of the last follow‐up the vast majority of patients presented with stable or decreased MBM. Only 7% of patients (11/151, 7%) were diagnosed with radiological tumor progression/ recurrence of at least one previously treated MBM. Three of these patients underwent microsurgical resection of their progressive MBM (3/151, 2%). The major aim of our study was to report the frequencies of radiation reaction/ necrosis and/or hemorrhage after GKRS in dependency of concomitant or prior to IT/TT. The majority of patients (114/151, 76%) showed no sign of intra‐tumoral hemorrhage after GKRS. In 20 patients (20/151, 13%), intralesional hemorrhage was diagnosed, but no therapy was deemed necessary.

In 17 (17/151, 11%) patients, symptomatic therapy, usually antiepileptic drugs, was prescribed. Three patients presented with space‐occupying and symptomatic hemorrhages after GKRS during IT/TT therapy. In all three patients, no surgical hematoma/ MBM evacuation was performed due to the patients’ bad clinical condition under the rapid peripheral progression.

**Figure 2:**
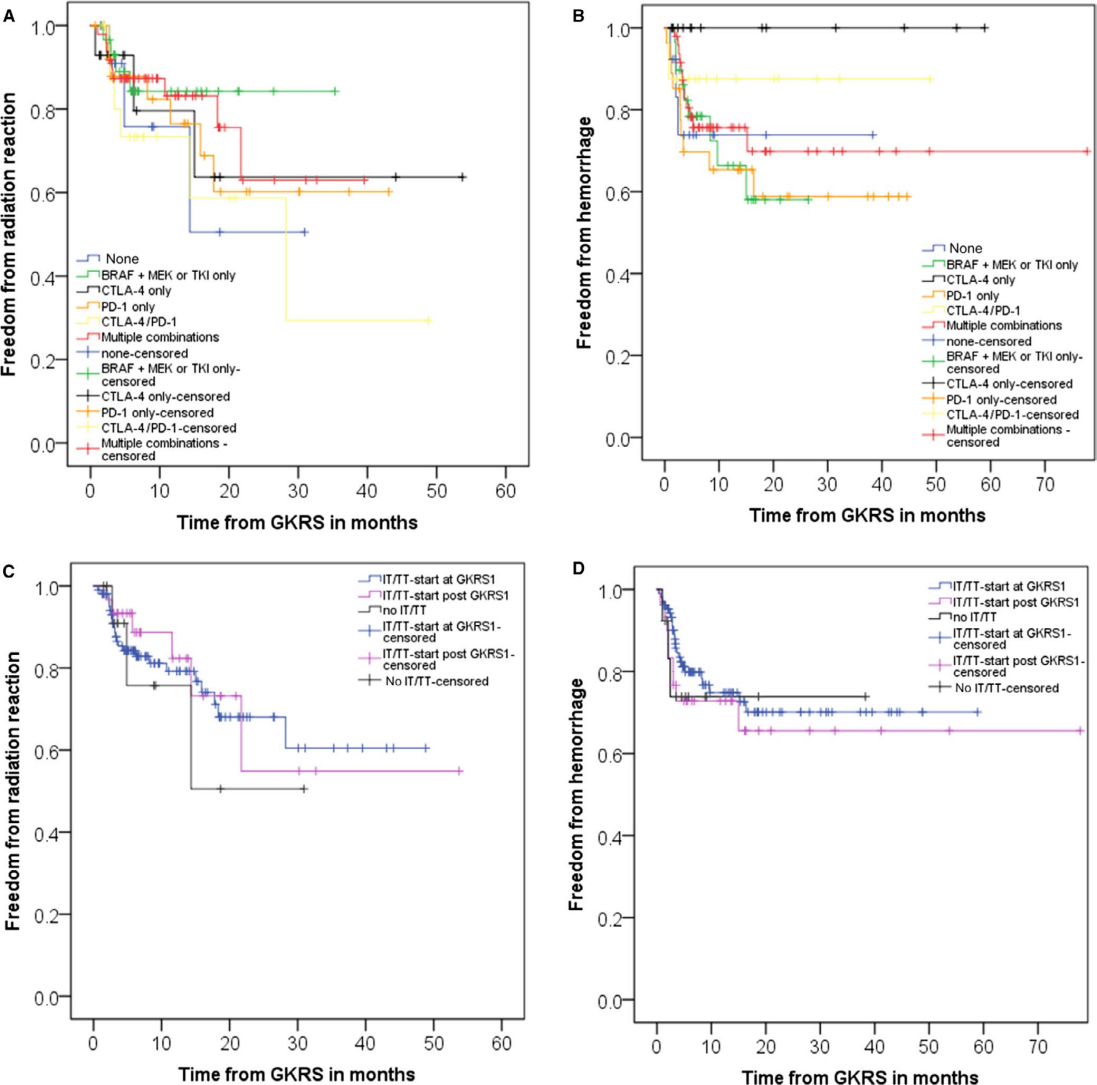
Occurrence of complications after GKRS1 in relation to modern oncological therapy. Figure shows the occurrence of complications after GKRS1 in relation to modern oncological therapy in 148 follow‐up patients for whom sufficient radiological and clinical follow‐up was available. A, represents the occurrence of radiation reaction after GKRS1 separated for the different IT/TT subgroups. The estimated median time to occurrence of radiation reaction did not show any significant differences between the different IT/TT subgroups (*P* = .891). As shown in B, the occurrence of hemorrhage after GKRS did also not differ between the IT/TT subgroups (*P* = .309). Interestingly, in those patients (14/148, 9%) who were treated with CTLA‐4 only, no hemorrhage could be observed. C, displays the occurrence of radiation reaction in relation to the different timing of IT/TT at and after GKRS1. The comparison between patients who were treated with IT/TT at or after GKRS and patients without IT/TT did not show any differences (*P* = .738). The estimated mean time of occurrence (median not reached) was longer in patients with IT/TT after GKRS1 (35.9 mo, 95% CI: 23.3‐48.6) than patients with IT/TT at GKRS1 (34.4 mo, 95% CI: 29.1‐39.8) or patients without IT/TT (20.2 mo, 95%: 10.9‐29.5). D, shows the same analysis for intralesional hemorrhage. This sub‐analysis did also not differ between the different timing of IT/TT (*P* = .719). The estimated mean time of occurrence (median not reached) of hemorrhage seems to be longer in patients with IT/TT after GKRS1 (52.7 mo, 95% CI: 38.6‐66.7) than patients with IT/TT at GKRS1 (43.1 mo, 95% CI: 37.5‐48.7) or patients without IT/TT (28.8 mo, 95%: 19.5‐38.1)

The majority of patients with radiological follow‐up (116/151, 78%) never suffered from a radiation reaction/ necrosis. However, in 22% of patients (33/151), a radiation reaction was noted at least for one MBM until the last follow‐up. Half of these patients presented with clinical symptoms and thus required corticosteroid treatment (17/151, 11%). The occurrence of hemorrhage or radiation reaction/necrosis after GKRS1 in relation to IT/TT did not show any statistically significant differences neither when analyzing IT/TT at time of GKRS1 nor when analyzing IT/TT ever after GKRS1 (Table 2). We did observe a nonsignificant trend of more intralesional hemorrhages after GKRS in patients treated with anti‐PD1 alone as well as a higher rate of radiation reaction/necrosis in patients treated with a combination of anti‐CTLA‐4/PD1 (Table 2). Next, we analyzed the timing of adverse reactions separated for IT/TT subgroups (Figure 2A,B) and in relation to the beginning of IT/TT, at the time of GKRS1 or > 30 days after GKRS1 (Figure 2C,D). Neither of those sub‐analyses revealed any significant differences.

**Table 2:** Radiologically diagnosed hemorrhage or radiation reaction/necrosis after GKRS1 in relation to IT/TT

IT/TT at or ever after GKRS1 n = 148	Hemorrhage ever after GKRS1 n = 148		Radiation reaction/necrosis ever after GKRS1 n = 148		
	None n/ %	Yes n/ %	None n/ %	Yes, w/o therapy n/ %	Yes, with therapy n/ %
None n = 13	10/ 77%	3/ 23%	10/ 77%	3/ 23%	0/ 0%
BRAF/MEK or TKI only n = 30	21/ 70%	9/ 30%	26/ 86%	2/ 7%	2/ 7%
Anti‐CTLA‐4 only n = 14	14/ 100%	0/ 0%	11/ 79%	1/ 7%	2/ 14%
Anti‐PD1 only n = 27	17/ 63%	10/ 37%	20/ 74%	5/ 19%	2/ 7%
Anti‐CTLA‐4/ PD1 n = 16	14/ 88%	2/ 12%	10/ 62%	3/ 19%	3/ 19%
Multiple combinations n = 48	36/ 75%	12/ 25%	39/ 81%	3/ 6%	6/ 13%
**Total**	**112/ 76%**	**36/ 24%**	**116/ 78%**	**17/ 12%**	**15/ 10%**

Note

The Table gives an overview of radiologically diagnosed complications after radiosurgery in 148 follow‐up patients for whom sufficient radiological and clinical follow‐up on IT/TT treatment was available. IT/TT are depicted in a pooled group combining a therapy start at and after GKRS1. Differences in the occurrence of complications in relation to the beginning of IT/TT are depicted in Figure 2C,D. Complications after radiosurgery were evaluated radiologically on follow‐up MRIs per patient. Thus, every new or progressive edema or intralesional hemorrhage even of a single BM was rated even if patients did not suffer from clinical symptoms. A progressing edema/ radiation reaction was treated with corticosteroids if patients developed symptoms. “Therapy” as shown in this table summarizes corticosteroid treatment but also all other forms of symptomatic treatments. Overall there is no statistically significant difference in the occurrence of complications in association with IT/TT although we did observe several trends. Statistical testing included overall cross tabs and chi‐square tests. Separate analyses for hemorrhages, radiation reaction/necrosis without therapy and with therapy as well as a pooled group of radiation reaction/ necrosis were performed. In addition, we performed pairwise chi‐square tests for each IT/TT subgroup versus “none” group. Neither of those analyses revealed any significant differences.

### 3.3 Time to new brain metastases and survival after radiosurgery in relation to immunotherapy and targeted therapy

**Figure 3:**
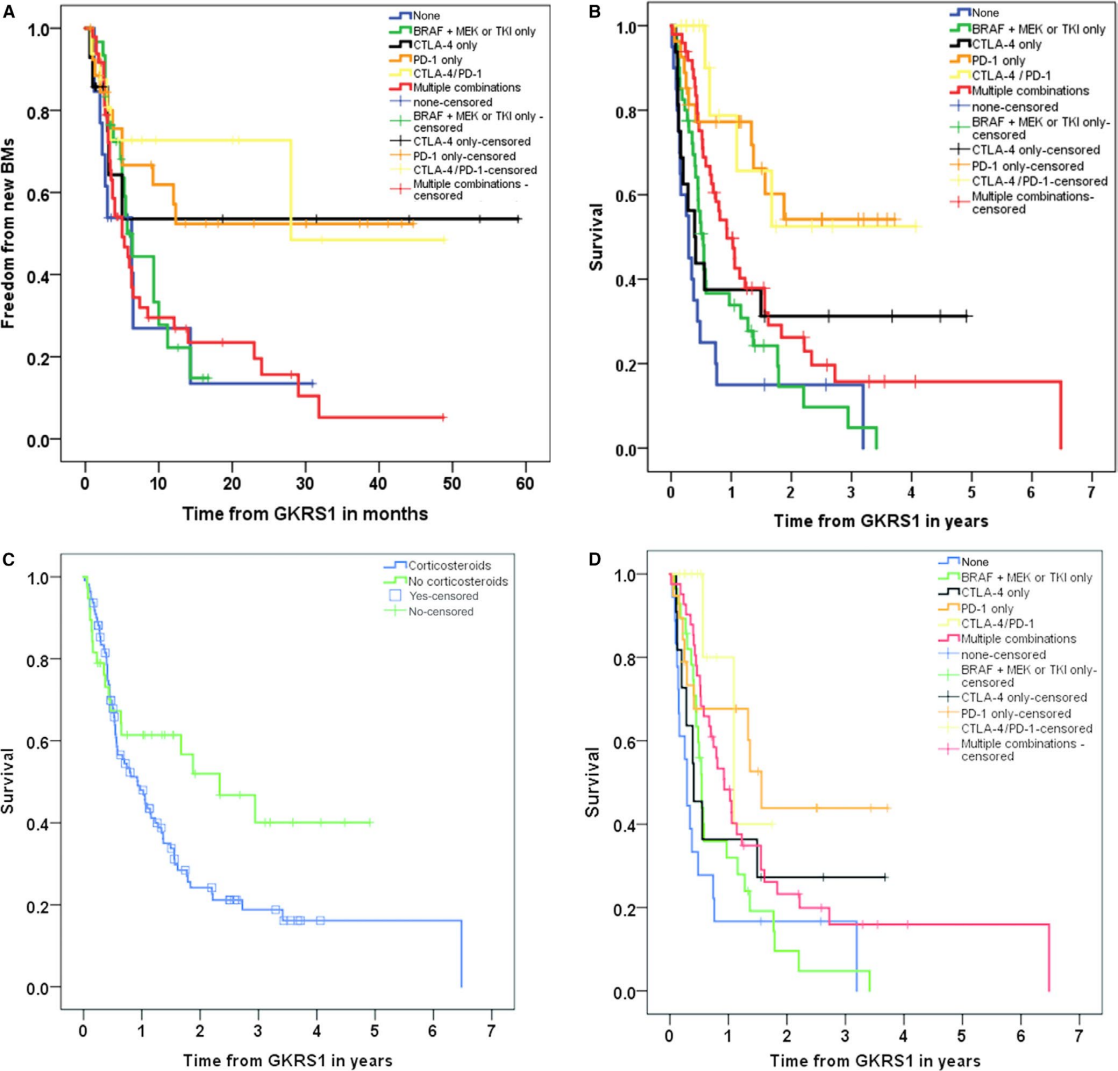
Time to new brain metastases and survival after radiosurgery in relation to modern oncological therapy. A, depicts time to new MBM after GKRS1 in months in 148 follow‐up patients for which a radiological follow‐up was available in relation to IT/TT. Patients who were not treated with any IT or TT developed new MBM in a median time of 6.3 mo (95% CI: 1.1‐11.5). Patients who received only BRAF and MEK inhibitors or TKI were diagnosed with new MBM after median time of 5.7 mo (95% CI: 4.2‐7.2). In the patient cohort treated with multiple combinations, the median time for developing new MBM after GKRS1 was 5 mo (95% CI: 2.8‐7.2).The most promising results were achieved for different checkpoint inhibitors (anti‐CTLA‐4: median not reached, mean = 32.9 mo, 95% CI: 15.6‐50.2; anti‐PD‐1: median not reached, mean = 25.9 mo, 95% CI: 17.8‐34.1 and anti‐CTLA‐4/PD1: median = 28.0 mo, 95% CI not calculable, mean 95% CI: 18.9‐43.3). B, shows survival after GKRS1 for different IT/TT subgroups in 168 follow‐up patients. Patients treated with anti‐PD‐1 (median not reached, mean = 2.4 y, 95% CI: 1.8‐3.0) or a combination of anti‐CTLA‐4/PD‐1 (median not reached, mean = 2.6 y, 95% CI: 1.6‐3.7) showed a significantly longer survival after GKRS1 compared to patients treated with anti‐CTLA‐4 only (median = 0.4 y, 95% CI: 0.1‐0.6) or other forms and combinations of IT/TT (multiple combinations: median = 0.9 y, 95% CI: 0.6‐1.3; BRAF + MEK or TKI: median = 0.5 y, 95% CI: 0.4‐0.6)) or no IT/TT at all (median = 0.3 y, 95% CI: 0.2‐0.4) (*P* < .001). C, presents differences in survival for 148 patients who received IT/TT at or after GKRS1 separated into patients treated and not treated with corticosteroids at or after GKRS1. Patients who received IT/TT after GKRS1 and were additionally treated with corticosteroids had a significantly shorter survival (median = 0.9 y, 95% CI: 0.6‐1.3) than patients who did not need corticosteroids (median = 2.3 y, 95% CI: 0.9‐3.8; *P *= .042). In a further sub‐analysis D, depicts survival after GKRS1 for different IT/TT subgroups *only* in those 128 follow‐up patients who received corticosteroids at or after GKRS1 for various reasons. Even among those patients treated with corticosteroids, the differences among IT/TT subgroups remain significant: Patients who did not receive any IT or TT show the shortest overall survival (median = 0.3 y, 95% CI: 0.2‐0.4), followed by patients treated with BRAF + MEK or TKI at or after GKRS1 (median = 0.5 y, 95% CI: 0.5‐0.6) or multiple combinations of IT/TT (median = 0.9 y, 95% CI: 0.6‐1.2) and patients treated with anti‐CTLA‐4 alone (median = 0.5 y, 95% CI: 0.1‐0.7). In contrast, treatment with anti‐PD‐1 (median = 1.6 y, 95% CI: 1.2‐1.9) or anti‐CTLA‐4/PD‐1 (median = 1.1 y, 95% CI: 0.3‐1.9) resulted in the best outcome after GKRS1 even among this subgroup. GKRS, Gamma Knife radiosurgery; IT, immunotherapy; MBM, melanoma brain metastases; TT, targeted therapy

As we have mentioned above, our local tumor control rate of GKRS‐treated MBM was 93% at last follow‐up. In contrast to the local tumor control rate, 59% of our follow‐up patients (89/ 151), were diagnosed with new, thus far untreated MBM (distant failure), after GKRS1. Consequently, 76% (67/ 89) of those patients received multiple GKRS treatments for new MBM. The remaining patients either refused or did not receive further treatment due to their clinical condition (13/89, 15%). Nine percent (8/89) of those patients were treated by WBRT as second line treatment for multiple MBM. The Kaplan‐Meier estimated the median time to new MBM was 6 months (95% CI: 3.9‐8.7 months). Patients treated with checkpoint inhibitors (anti‐PD‐1, anti‐CTLA‐4, or a combination of anti‐CTLA‐4/PD‐1) showed a significantly longer time to new MBM after GKRS1 compared to patients treated with other forms and combinations of IT/TT or no IT/TT at all (*P *= .012; Figure 3A).

Overall, the estimated median survival after the initial diagnosis of MBM was 1.0 year (95% CI = 0.7‐1.2 years) and 0.8 years (95% CI = 0.4‐1.1 years) after first GKRS. There were no significant differences among melanoma subtypes regarding survival after the initial MBM diagnosis or first GKRS treatment. In contrast, survival times in our cohort were significantly longer compared to the calculated prognostic survival times according to the general GPA (*P* < .001), specific GPA (*P *< .001), RPA (*P* < .001), and SIR (*P* < .001).

Significant differences in survival GKRS1 were observed when comparing different oncological therapies. Patients treated with anti‐PD‐1 or a combination of anti‐CTLA‐4/PD‐1 showed a significantly longer survival after GKRS compared to patients treated with other forms and combinations of IT/TT or no IT/TT at all (*P *< .001, Figure 3B). This significantly longer survival remains significant even when only patients with a KPS >= 80 are included (*P *= .006, data not shown). Of note, among this subgroup patients treated with anti‐CTLA‐4 only additionally show a benefit in survival after GKRS1.

Overall, the majority of patients (134/176, 76%) received corticosteroids at or after GKRS1. Fifty‐four patients (54/176, 31%) were treated with corticosteroids at the time of GKRS due to the neurological symptoms at MBM diagnosis. Consequently, corticosteroids were tapered over the next few weeks. Still, 80 patients (80/176, 45%) additionally received corticosteroids more than 30 days after GKRS1. The underlying reasons for additional corticosteroid treatment ranged from the occurrence of new MBM to toxicity of their oncological therapy or symptomatic radiation reactions as described above. A simple subgroup analysis revealed that patients who received IT/TT after GKRS1 and were additionally treated with corticosteroids had a significantly shorter survival (*P *= .042, Figure 3C). Of note, when including only those patients who *did* receive corticosteroids at or after GKRS1, the above described difference in survival among treatment groups remained significant (*P *= .004, Figure 3D).

## 4 DISCUSSION

### 4.1 Patient presentation and overall survival

Historically, melanoma patients had a poor prognosis.^18^ Recent developments in new oncological therapies have led to a longer overall survival seem to have a positive effect on MBM.^19^, ^20^, ^21^ In our present study, we report a median overall survival of 5.4 years after the initial diagnosis of melanoma, which is similar to or higher than in other studies.^19^, ^22^ The distribution of melanoma subtypes, BRAF status, and baseline clinical characteristics of our study patients (Table 1) comprise a representative cohort of MBM patients.^21^, ^23^ Nevertheless, we would like to comment on our KPS range at the time of the first GKRS: at our institution, patients in palliative settings are also treated if a benefit from the treatment might be anticipated. Those decisions are always made according to the patients’ wishes and in interdisciplinary agreement of the radiosurgeon and the dermato‐oncologist.

### 4.2 Local tumor control and radiological follow‐up after radiosurgery in relation to immunotherapy and targeted therapy

Overall, local tumor control rates (LCR) of 81%‐95% after radiosurgical MBM treatment have been reported with an apparent improvement in the modern oncological and radiosurgical era.^22^, ^24^, ^25^, ^26^, ^27^ In our study population, a local tumor control rate of 93% could be observed, representing our modern treatment era. However, the results regarding the effect of IT and TT on LCR are controversial. Recent studies reported no differences in the local tumor control of irradiated MBM among different IT/TT treatment groups or based on BRAF status.^28^, ^29^ Irrespective of the effect that modern oncological therapies seem to have on LCRs for MBM, the even more prominent question that has not been sufficiently addressed is about possible side effects of concurrent radiotherapy or radiosurgery and IT or TT. A recent review concluded that cranial SRT was mostly well tolerated in combination with the majority of targeted drugs and IT but the combination with BRAF‐inhibitors should be practiced with caution due to severe toxicity events.^30^ Specific toxicity data on MBM and radiosurgery with concurrent IT/TT are sparse.

MBM are generally known to have a high disposition for intralesional hemorrhage, with rates of up to 50% even before the first specific treatment.^18^, ^31^, ^32^ Recent studies reporting on the combined treatment of anti‐PD1 or anti‐CTLA‐4 and stereotactic radiosurgery (SRS), did not show an increased hemorrhage rate.^21^, ^28^, ^33^, ^34^ Other studies described an increased hemorrhage rate in patients treated with SRS and BRAF inhibitors despite the reported improvement in local control.^35^ Overall, in our study, we observed intralesional hemorrhage in roughly a quarter of our patients after GKRS, which is in line with previous studies prior to the IT/TT era.^18^, ^31^, ^32^

Besides hemorrhage, radiation necrosis (RN) or adverse radiation effects (ARE) are well‐known complications after radiosurgery and can occur in up to 24%, which is comparable to our rate of 22%.^36^, ^37^ In contrast, several recent studies reported on slightly higher rates of RN/ARE in patients treated with IT or TT.^26^, ^38^, ^39^ Other recent studies even reported an association between the receipt of IT and symptomatic or late radiation necrosis.^40^, ^41^ In our study, the occurrence of hemorrhage or radiation reaction/necrosis after GKRS1 in relation to IT/TT did not show any statistically significant differences neither when analyzing IT/TT treatment starting at the time of GKRS1 nor when analyzing IT/TT treatment starting > 30 days after GKRS1. We only observed a not significant trend of more intralesional hemorrhage after GKRS in patients treated with anti‐PD‐1 alone as well as a not statistically significant higher rate of radiation reaction/necrosis in patients treated with a combination of anti‐CTLA‐4/anti‐PD‐1. Still, these contradicting results, so far reported in the literature, warrant further studies. However, we would like to comment that some of the publications reporting on an increased risk of adverse reactions after radiotherapy/radiosurgery in combination with IT or TT do not specifically state their radiosurgical technique or seem to report on a heterogeneous cohort of patients treated with radiosurgery and fractionated radiotherapy.^39^, ^40^, ^41^

### 4.3 Time to new metastases and survival after radiosurgery in relation to immunotherapy and targeted therapy

Historically, the survival of melanoma patients after the initial diagnosis of brain metastases was short. Local therapies such as surgical resection or radiosurgery significantly improved the survival after MBM diagnosis.^42^, ^43^ The advent of new oncological therapies further increased the overall survival of melanoma patients.^28^, ^38^, ^44^ Recent studies on brain metastases, albeit not specific for melanoma patients, showed a positive effect of concurrent checkpoint inhibitors and SRS for brain metastases on overall survival and on local progression‐free survival, but not on distant progression‐free survival.^45^ More specifically, a recent study on MBM and SRS revealed improvement in distant MBM control with anti‐PD‐1 therapy and BRAF/MEKi regimens over conventional chemotherapy.^28^

In our study, the benefit of IT on distant control was even more pronounced. We observed a clear and statistically significant improvement in distant control after GKRS in MBM patients who received anti‐PD‐1, anti‐CTLA‐4, or a combination of anti‐PD‐1/anti‐CTLA‐4 at or after GKRS, in comparison to conventional therapy or BRAF/MEKi. In addition, our patients treated with checkpoint inhibitors, including anti‐PD‐1 or anti‐PD‐1/anti‐CTLA‐4, also showed more favorable results in survival after GKRS1, compared to other forms of the oncological therapy.

Recent studies reported on a significantly longer overall survival in melanoma patients who were treated with the combination of anti‐PD‐1/anti‐CTLA‐4, or with anti‐PD‐1 alone, compared to those treated with anti‐CTLA‐4 alone. Furthermore, the combination of anti‐PD‐1/anti‐CTLA‐4 has shown cranial response rates of up to 50%.^46^ The incidence of treatment‐related adverse events has been reported to be significantly higher with combination therapy than with anti‐PD‐1 or anti‐CTLA‐4 alone. In our study, we did not observe a difference in regard to distant control and survival in MBM patients after radiosurgery between anti‐PD‐1 alone and the more toxic combination of anti‐PD‐1/anti‐CTLA‐4 treatment. In contrast to previous studies, these findings represent the survival from the first radiosurgical treatment of brain metastases and not the overall survival from melanoma diagnosis.^46^

Moreover, glucocorticoids have been discussed to diminish the effect of IT due to their immunosuppressive activity.^47^ In our sample, an overall reduction of survival time was also evident in patients who received glucocorticoids. Since this analysis might be severely biased due to various indications for corticosteroid treatment, we performed a different sub‐analysis. Even when including only those patients who *did* receive corticosteroids at or after GKRS1, the above described differences in survival among treatment groups remained significant.

### 4.4 Limitations of our study

Limitations of our study include its retrospective character and its center‐ and treatment‐biased nature. Furthermore, our endpoints and time intervals between the drug delivery and SRS were not predefined but rather covered ± 30 days at GKRS and the period from first radiosurgical treatment. Since the observation period of our study started with the first radiosurgical treatment, we do not differ between different oncological pretreatments prior to the diagnosis of MBM, which may introduce a selection bias. This was done to evaluate concomitant IT or TT and GKRS at time of or after first radiosurgical treatment for MBM irrespective of prior treatments. As others have described before, we retrospectively evaluated complications after radiosurgery on serial standard follow‐up MRIs and according to the RANO criteria. Still, in only some of our patients PET‐MRI or perfusion sequences were available. Thus, the correct evaluation of RN or true progression remained a challenge.

## 5 CONCLUSION

According to our data, complications after GKRS in MBM patients, as defined by hemorrhage and radiation reaction/necrosis, are not significantly increased if IT/TT treatment is performed at the time of or after GKRS1. Further, a clear benefit in distant control and survival after SRS is seen in MBM patients treated with GKRS and anti‐PD‐1 or a combination of anti‐PD‐1/anti‐CTLA‐4. Thus, concomitant treatment of MBM with GKRS and IT/TT seems to be a safe and powerful treatment option although further prospective studies should be conducted.

## CONFLICTS OF INTEREST

None of the authors disclosed any competing interests or specific funding regarding this retrospective study.

## AUTHOR CONTRIBUTIONS

Brigitte Gatterbauer: Data acquisition, data analysis, interpretation, validation, writing—original draft, and editing. Dorian Hirschmann: Data acquisition, data analysis, interpretation, writing—original draft, and editing. Nadine Eberherr: Data acquisition, data analysis, interpretation, writing—review and editing. Helena Untersteiner: Data acquisition, data analysis, interpretation writing—review and editing. Anna Cho: Data analysis, interpretation, writing—review and editing. Abdallah Shaltout: Data acquisition, interpretation, writing—review and editing. Philipp Göbl: Data analysis, interpretation, writing—review and editing. Fabian Fitschek: Data acquisition, interpretation, writing—review and editing. Christian Dorfer: Data acquisition, validation, writing—review and editing. Stefan Wolfsberger: Data acquisition, validation, writing—review and editing. Gregor Kasprian: Data interpretation, validation, writing—review and editing. Christoph Höller: Conceptualization, data acquisition, interpretation, validation, writing—review and editing. Josa M. Frischer: Conceptualization, data acquisition, data analysis, interpretation, supervision, validation, writing—original draft, and editing.

## Data Availability

Research data are not shared.
